# Wakeful rest and memory consolidation in an ecologically valid educational setting: no benefit over distractor tasks

**DOI:** 10.1007/s00426-026-02265-x

**Published:** 2026-02-26

**Authors:** Peter Seban, Radovan Šikl, Tomáš Prošek, Kamila Urban

**Affiliations:** 1 Institute for Research in Social Communication, Slovak Academy of Sciences, Dúbravská cesta 9, Bratislava, 841 04 Slovakia; 2 Institute of Psychology, Czech Academy of Sciences, Prague, Czech Republic

**Keywords:** wakeful resting, distractor task, social media use, similarity paradox, text comprehension

## Abstract

Post-learning activities are thought to influence how well newly learned information is consolidated into memory. The present study investigated whether wakeful rest provides advantages for memory consolidation compared to common distractor activities. University students (*N* = 161) read an expository text and were randomly assigned to one of four 8-minute post-reading conditions: wakeful rest, social media use, math task, or reading an interference text. Participants completed an immediate test assessing both conceptual understanding and factual recall, followed by a delayed test one week later using different but conceptually aligned test items. Results showed a significant decline in performance from the immediate to the delayed test across all groups. However, the wakeful rest condition did not yield consistent benefits relative to the distractor conditions. These findings suggest that in a real-world educational setting, wakeful rest may not provide superior benefits for conceptual understanding and memory retention relative to other post-learning activities.

Effective learning depends critically on robust memory consolidation, a time-dependent process that transforms newly acquired information into stable, long-term representations (Wixted & Cai, 2014). This process safeguards recently encoded memories from interference and ensures their accessibility over time (Dudai et al., 2015; Robertson, 2012; Wixted, 2004; Wixted & Cai, 2014). Neuroscientific research suggests that heightened post-learning activity in brain regions involved in initial encoding contributes to memory stabilization and supports delayed recall (Tambini et al., 2010; Wixted, 2004). A central mechanism underlying consolidation is neural replay, the reactivation of memory traces during sleep or periods of quiet rest, which facilitates their integration and long-term storage (Carr et al., 2011; Dudai et al., 2015; Wamsley, 2022). This replay-based account is consistent with behavioral findings showing improved memory performance following periods of sleep or rest, when sensory input and cognitive demands are reduced (Foster & Wilson, 2006; Wamsley, 2022; Wilson & McNaughton, 1994).

While much of the literature has focused on sleep-dependent consolidation, emerging evidence highlights the role of offline wakeful states, particularly wakeful rest (WR), in supporting memory retention (Weng et al., 2025). WR refers to a state of relaxed wakefulness in which a person is awake but not actively engaged in any demanding cognitive or physical task. During this period, external attention is reduced, allowing the brain to enter an “offline” mode that supports memory consolidation (Martini et al., 2022; Wamsley, 2019). Research indicates that such states can facilitate memory consolidation by reducing cognitive interference immediately after learning (Craig & Dewar, 2018; Craig et al., 2016). These periods are thought to provide a “quiet” window during which newly formed memories are stabilized, making them less susceptible to decay or disruption.

Importantly, evidence for a WR benefit has emerged when WR is contrasted with post-learning activities that impose cognitive, perceptual, or executive demands (Martini & Sachse, 2020). Across the literature, such distractor conditions have included numerical tasks (e.g., n-back tasks: Horlyck et al., 2019; Varma et al., 2017; digit monitoring tasks: Millar & Balota, 2022), visuospatial problem solving (Martini et al. 2018, 2019a), spot-the-difference tasks (Dewar et al., 2014; Leetham et al., 2024; Sacripante et al., 2019), snood tasks (Brokaw et al., 2016; Wang et al., 2021), picture search (Craig et al., 2014), psychometric tests (Cowan et al., 2004), video watching (Martini et al., 2021), reading in a second language (Martini et al. 2020b), listening to music (Martini et al., 2022), and social media use (Martini et al. 2020a).

Moreover, WR itself is not implemented uniformly across studies. In most paradigms, WR typically involves sitting quietly with eyes closed (Martini et al. 2020b), open (King & Nicosia, 2022), with no limitations (Varma et al., 2017), or without explicit instructions (Sacripante et al., 2019). Taken together, this variability in both WR and distractor implementations suggests that observed WR effects may depend critically on the specific cognitive, attentional, and perceptual demands imposed during the post-learning interval, rather than on a simple rest-versus-distraction distinction.

Consistent with this heterogeneity in both wakeful rest and distractor implementations, the beneficial effects of WR are not always robust or universally observed. Recent work suggests that WR efficacy depends on multiple moderating factors, including the nature of the post-learning task to which it is compared, the characteristics of the learned material, and the level of cognitive engagement following encoding (Martini & Sachse, 2020; Weng et al., 2025). In particular, distractor tasks that draw on cognitive resources largely distinct from those involved in the original learning episode may exert only minimal interference on consolidation processes. This “minimal overlap” account posits that post-learning activities engaging different cognitive systems are less likely to disrupt newly formed memory traces (Varma et al., 2017, 2018). Accordingly, empirical studies have shown that even cognitively demanding activities (e.g., n-back tasks) or tasks involving language learning do not consistently reduce retention relative to WR, underscoring the importance of task characteristics, representational overlap, and material complexity in shaping post-encoding outcomes (Martini et al. 2020b; Varma et al. 2017, 2018).

Furthermore, recent efforts to examine WR in more naturalistic or applied settings have produced mixed results. For instance, Brandmark et al. (2020) investigated WR in a classroom-like environment in which participants viewed an educational video and were later tested using a multiple-choice knowledge test. Their findings showed no significant differences in either immediate or delayed memory performance between the WR and distractor groups. Similarly, Leetham et al. (2024) conducted an online study to explore WR in real-world contexts and found that participants often had difficulty maintaining a restful state. This lack of adherence to the intended cognitive state may have contributed to the absence of memory benefits. Together, these findings raise important questions about the generalizability of WR effects outside of controlled laboratory conditions and underscore the challenges of implementing WR in more ecologically valid learning contexts.

## Present study

The goal of the present study is to examine whether WR supports comprehension and long-term retention of an expository text, and to compare its effects with post-learning activities that differ in cognitive and verbal demands. By focusing on comprehension of written material, this study extends prior WR research that has primarily emphasized recall of word lists or visuospatial tasks (Parra et al., 2026; Weng et al., 2025). In doing so, it addresses a question of particular relevance for educational practice, where understanding and retaining complex texts is central.

In addition to comprehension and long-term retention, we also assess recall performance, which has been the dominant focus in earlier WR studies. Participants were randomly assigned to one of four post-learning conditions: (1) WR (implemented through autogenic training), (2) social media use, (3) solving mathematical problems, or (4) reading a second, content-similar text. Each condition was chosen for its theoretical and practical significance to cognitive and educational research:

### Autogenic Training as WR

WR has been criticized for its vulnerability to mind-wandering, which can reduce memory performance (Leetham et al., 2024; Varma et al., 2018), or, conversely, for potentially allowing rehearsal of learned content. To minimize both risks, we employed autogenic training, a guided relaxation technique designed to help participants maintain a relaxed state while reducing spontaneous rehearsal (Leetham et al., 2024).

### Social Media Use

Social media platforms are integral to daily life but, in general, have been consistently associated with poorer academic performance and impaired memory outcomes in educational contexts (Frein et al., 2013; Junco, 2012; Kirschner & Karpinski, 2010; Spence et al., 2020). Within the wakeful rest literature, Martini et al. (2020a) directly compared post-learning wakeful rest with social media use and found that social media usage led to poorer recall performance than wakeful rest, both immediately after the post-learning interval and after a 1-day delay. These findings suggest that the sustained sensory and attentional engagement characteristic of social media use may interfere with post-encoding consolidation processes.

### Mathematical Problems

Math-based activities are commonly used as post-learning distractor tasks in memory research, as they require sustained cognitive processing while remaining largely unrelated to the representational content of verbal learning materials. Basic arithmetic relies on domain-general executive and numerical processes and therefore minimizes semantic or conceptual overlap with text comprehension or verbal memory representations (Glenberg et al., 1980; Peterson & Peterson, 1959). For this reason, arithmetic problem solving has a long tradition as a neutral distractor used to fill retention intervals and prevent rehearsal without inducing similarity-based interference, while reliably occupying cognitive resources during the post-learning interval (Dewar et al., 2007; Millar & Balota, 2022; Varma et al., 2017).

### Content-similar reading passage

Reading a second, content-related text constitutes a theoretically ambiguous post-learning activity. On the one hand, engaging with semantically related material may introduce similarity-based interference, whereby overlapping representations compete and increase the risk of forgetting (McGeoch, 1931; Wixted, 2004). On the other hand, exposure to related information may promote elaborative processing, conceptual integration, and schema construction, which are known to support comprehension and long-term retention (Kintsch, 1988, 1998). Also, research on interference suggests that greater similarity does not necessarily lead to greater forgetting. As articulated in the similarity paradox (Osgood, 1949), highly similar material can sometimes reduce interference by facilitating integration or discrimination rather than competition.

In summary, the three distractor conditions were selected to represent theoretically distinct forms of post-learning interference. Social media use reflects an ecologically valid post-learning activity characterized by sustained attentional capture, rapid content switching, and continuous sensory input, all of which are assumed to interfere with consolidation processes (Martini et al. 2020a). Solving mathematical problems represents a cognitively demanding but semantically unrelated distractor, commonly used in memory research to occupy cognitive resources while minimizing content overlap with verbal learning materials (Peterson & Peterson, 1959). Finally, reading a content-similar text constitutes a theoretically ambiguous condition: while semantic similarity may induce retroactive interference and impair retention (Wixted, 2004), it may also promote elaboration, or conceptual integration (Kintsch, 1998). Wakeful rest was therefore expected to provide particularly favorable conditions for consolidation by minimizing post-encoding interference, particularly relative to attention-demanding and cognitively engaging distractor activities.

Based on interference-based accounts of memory consolidation, we hypothesized that wakeful rest would lead to better immediate (H1a) and delayed (H1b) knowledge test performance than post-learning activities that involve sustained attentional engagement or cognitive load (e.g., social media use and mathematical problem solving). Performance following the content-similar reading condition was treated as an open empirical question, given competing predictions of retroactive interference versus elaborative integration.

For recall-based memory questions, we expected wakeful rest to yield better immediate (H2a) and delayed (H2b) performance relative to attentionally and cognitively demanding distractor tasks, while effects of content-similar reading remained exploratory.

## Methods

### Participants and design

A priori power analysis was conducted using G*Power (version 3.1.9.7; Faul et al., 2007) for detecting a between-subjects main effect in a repeated-measures design. Assuming a medium effect size (*f* = 0.25), α = 0.05, and power = 0.80, a minimum of 140 participants (35 per group) was required.

A total of 180 university students (45 per group) initially participated in the study. Nineteen participants were excluded based on the following exclusion criteria: one reported a diagnosed reading impairment; four reported a first language different from the language of the experimental materials; five did not complete the final test; one was a student of biology; two had extensive prior knowledge of the topic (see below); and six indicated they were unable to concentrate on the autogenic training due to mind-wandering (assessed at the end of the first testing session via self-report item describing difficulty maintaining attention on the procedure because thoughts repeatedly shifted to unrelated thoughts or memories).

The final sample consisted of 161 undergraduate students (135 women, 24 men, 2 identifying as other; *M*_*age*_ = 21.97, *SD*_*age*_ = 1.75) from three state universities. The sample was homogeneous in terms of race and socioeconomic status. The study was approved by the Ethics Committee of the Slovak Academy of Sciences, under the number: 26042024. Participants received partial course credit for their participation.

**Fig. 1:**
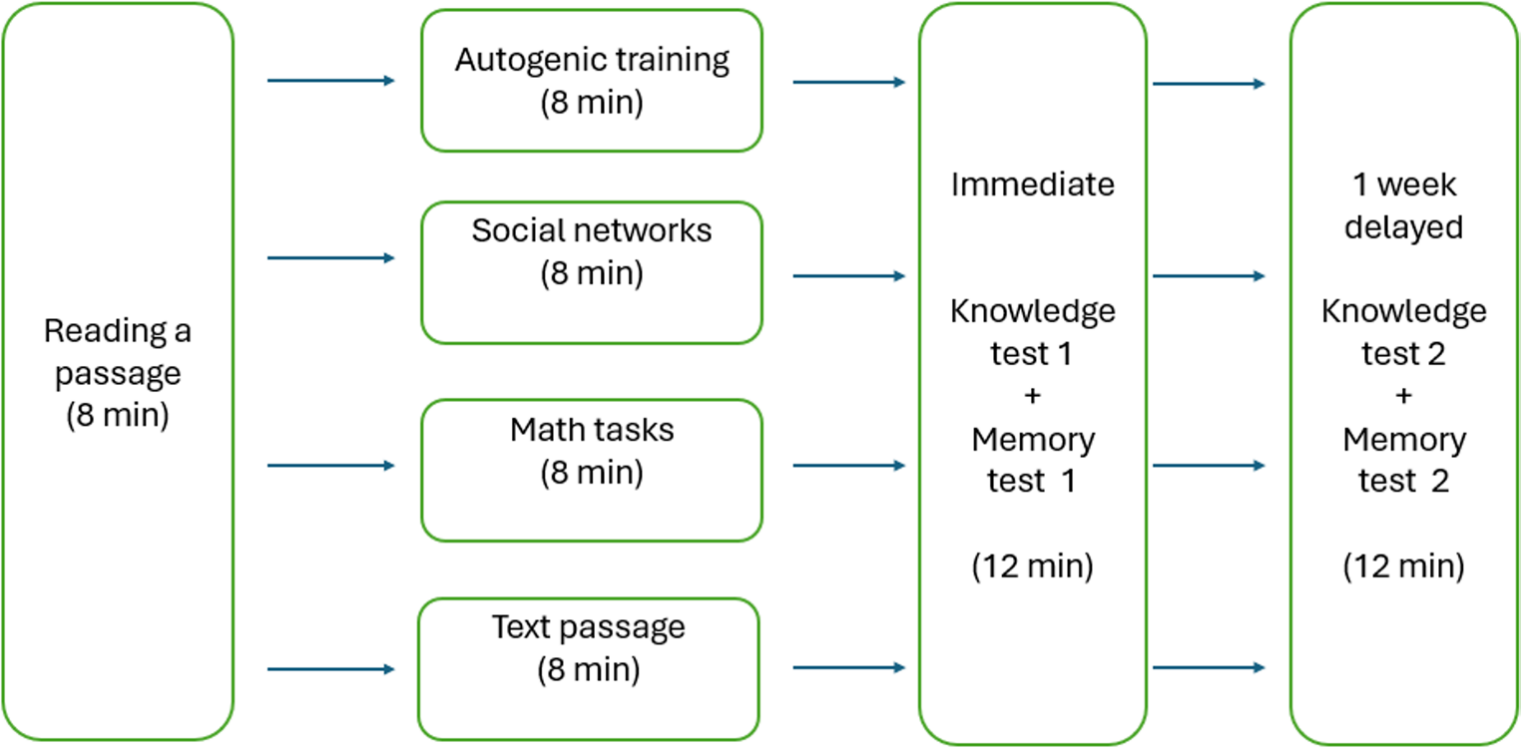
Sketch of the experimental procedure

Prior to the study, participants provided informed consent and were told that the research focused on text comprehension. They were informed of their right to withdraw at any time without explanation. To reduce bias, participants were not made aware of the specific hypotheses or aims of the study. Participants were randomly assigned to one of four experimental groups: wakeful resting – autogenic training, social media browsing, math task, or text reading (see Fig. 1).

### Materials

#### Prior knowledge test

To assess participants’ prior knowledge without inducing a pretesting effect, we used a single open-ended question administered before the reading phase: “What do you know about the pecking behavior of woodpeckers?” Participants’ responses were screened for content-relevant domain knowledge related to the biomechanical explanations presented in the experimental text. Vast majority of responses reflected only general, non-specific knowledge (e.g., that woodpeckers peck trees to obtain food or have specialized beaks) and showed minimal variability; these responses were coded as indicating no prior knowledge. Only two participants provided content-relevant explanations closely aligned with the material covered in the text (e.g., mentioning protective adaptations such as mechanisms reducing impact forces on the brain). These participants were therefore classified as having extensive prior knowledge and excluded from the analyses.

#### Main text

An expository text in the participants’ native language was used as the study material. It was selected for its unfamiliar topic, attractiveness, and ease of comprehension, and tailored to an 8-minute reading time. The text, titled *The Research on Woodpecker Pecking*, was 617 words long and presented scientific findings in an accessible, popular-science style. The Flesch reading ease score was 65.5.

The content explained how woodpeckers can peck without sustaining brain damage, focusing on head size, biomechanical adaptations, and differing scientific perspectives on the phenomenon. The text did not include any typographic or visual signaling (e.g., boldface, italics, headings, or bullet points) to highlight important information.

Participants were instructed to read the text carefully for the full 8 min, to reread if necessary, and to focus on comprehending the information. Prior to reading, they were informed that the final test would assess their understanding of the material, with particular emphasis on conceptual comprehension and the explanation of key ideas.

#### Knowledge test 1 (immediate) and knowledge test 2 (delayed)

The knowledge test included five open-ended questions designed to assess participants’ comprehension of key concepts from the text. Each question required an explanatory constructed response. The immediate (Test 1) and delayed (Test 2) tests included different sets of questions. To ensure comparability and avoid order effects, a pilot study was conducted using a larger pool of potential questions. Based on this, ten questions were selected and divided into two equivalent sets of five, matched for conceptual coverage and estimated difficulty. Test 1 was administered at the immediate post-test and Test 2 at the delayed post-test for all participants.

##### Scoring

Each response could earn a maximum of two points, resulting in a total possible score of 10 points per test. Responses were independently evaluated by the first and second authors, both trained in cognitive and educational psychology. To minimize bias, both raters were blinded to participants’ group assignments. A research assistant anonymized all test materials before scoring. A detailed scoring rubric was developed prior to evaluation, outlining the criteria for awarding 0, 1, or 2 points per answer. The rubric aimed to ensure objectivity and consistency across raters. Both authors independently scored all responses using this rubric. Interrater reliability was high (Cohen’s κ = 0.88). Any discrepancies between raters were resolved through discussion until agreement was reached.

To summarize the principles of the scoring rubric with an example for the question *“How was the research on woodpecker pecking conducted?*”: Two points were awarded for a fully correct and sufficiently detailed response demonstrating clear understanding, e.g.: *" Research consisted of analyzing video footage of pecking of woodpecker*,* and quantification of the slowdown of the impact of pecking and creation of biomechanical models.”* One point was given for a correct response that showed some understanding but was either incomplete or excessively general, e.g., *“Researchers analyzed videos of pecking of 3 kinds of woodpeckers.”* Zero points were given for an incorrect or irrelevant response or no response at all, e.g.: *“Researchers were observing woodpeckers to peck to the trees.”* or *“They measured the anatomy of woodpeckers.”*

#### Memory test 1 (immediate) and memory test 2 (delayed)

Each memory test consisted of five open-ended questions assessing participants’ recall of specific factual details explicitly stated in the text. In contrast to the knowledge test, which targeted conceptual understanding and explanation, the memory test focused on verbatim or near-verbatim recall of discrete information (e.g., *“What is the size of the largest species of woodpeckers?”*; “*From which university did the scientists conducting the research come?”*). Participants could earn one point per correct answer, for a maximum score of five points per test. Scoring was binary: one point for accurate recall and zero points for incorrect or missing responses. All responses were evaluated by the first author using predefined scoring criteria. Given the objective nature of the questions (factual information with clearly defined correct answers), scoring was considered unambiguous; to ensure reliability, the last author independently checked a random subset of responses, confirming consistency in scoring across raters. Memory Test 1 and Memory Test 2 featured different question sets, selected based on a pilot study to ensure comparable difficulty and content coverage.

### Distractor Tasks

#### Autogenic training (Wakeful Resting)

Autogenic training is a passive, auto-suggestive relaxation technique that can be performed independently and requires minimal training. It consists of six mental exercises based on self-directed suggestions (McNeil & Lawrence, 2002; Schultz & Luthe, 1959). In this study, the intervention was delivered live by the first author, who read the guided instructions. Participants sat comfortably with their eyes closed and were instructed by the experimenter to breathe slowly and deeply.

The first part of the session focused on inducing a sensation of heaviness in the arms and legs through repeated verbal cues. In the second part, participants were guided to imagine a spreading warmth in their limbs and throughout their bodies. Throughout the training, participants were reminded to remain calm and relaxed. The session concluded by redirecting attention to their breathing. The full procedure lasted 8 min. Autogenic training was used to help participants remain mentally calm, to minimize mind wandering, and to prevent rehearsal.

#### Use of social networks

Participants assigned to this condition were instructed to use their mobile phones to scroll through their usual feed (Facebook or Instagram) at their own pace for 8 min. The experimenter unobtrusively monitored compliance by periodically walking around the classroom to ensure that participants were actively scrolling through their social media feeds.

#### Math task

In this condition, participants solved math problems involving basic arithmetic (addition, subtraction, multiplication, and division), including items of varying difficulty that combined these operations. Participants were instructed not to use calculators and were told that completing all math problems was not required. The task duration was 8 min.

#### Content similar reading passage

To parallel the reading activity in the experimental design while introducing conceptual overlap, we developed a control reading passage matched to the target text for length and reading time (8 min). This text, titled *“Tailorbirds*,*”* described the distinctive nest-building behavior of tailorbirds, including their sewing-like construction technique, the structure and appearance of completed nests, and ecological challenges such as predation and invasive species. The passage contained 635 words and had a comparable readability level (Flesch Reading Ease Score = 69.2).

### Procedure

All enrolled participants were randomly assigned to one of four experimental groups. Each group was then invited to a scheduled classroom session conducted under the experimenter’s direct supervision. Upon arrival, participants were informed that the study was anonymous and were asked to generate a personal code to allow the researchers to match data across sessions while preserving confidentiality. Participants were told that the final test would consist of two parts: (1) five open-ended questions assessing understanding and explanation of key concepts, and (2) five factual questions without answer options, focusing on the recall of specific information. Participants completed the tests by typing their responses on their personal laptops using a computer-based test battery implemented via SurveyMonkey.

The experimental procedure began with an eight-minute reading session. Participants were instructed to read the assigned text attentively, as if preparing for an exam. They were allowed to reread any part of the text during this time. Immediately after reading, participants underwent an 8-minute intervention specific to their assigned group (see section Distractor Tasks). This was followed by the immediate post-test, for which participants had 12 min to complete both the knowledge and memory components. At the end of the session, participants were asked not to read or study any material on woodpeckers over the coming week to avoid contamination of delayed test results.

One week later, participants returned at a prearranged time to complete the delayed test, which contained different - but comparable - items from the immediate post-test. Before starting the delayed test, participants completed a brief self-report item asking whether they had read or studied any materials related to woodpeckers during the intervening week; no participant reported doing so. After completing the second test, the researcher debriefed the participants, explained the purpose of the study, and expressed gratitude for their participation.

### Data analysis

Statistical inference analysis was conducted in JASP version 0.19.3 (JASP Team, 2025). Data have been made publicly available at the OSF repository and can be accessed at the link: https://osf.io/su9j7/overview?view_only=e5e32252bfa648a6894025ed36b07584.

All analyses were conducted using repeated-measures ANOVA. For each dependent variable, time of test (immediate vs. delayed) was entered as a within-subjects factor, and type of distraction task (four groups) as a between-subjects factor. All models included the interaction between these two factors. Assumptions of normality and homogeneity of residuals were assessed visually using Q–Q plots and residuals versus fitted values scatterplots (Field, 2005). Model stability was examined using Cook’s distance. No substantial violations of model assumptions or influential cases were detected, indicating that the models provided an adequate fit to the data.

## Results

Descriptive statistics (means and SEs) for measured variables are displayed in Table 1.

**Table 1:** Means and Standard Errors (SE) for measured variables – knowledge and memory tests

Time of the test	Experimental group	*N*	M_knowledge_ (%)	SE_knowledge_	M_memory_ (%)	SE_memory_
Immediate	WR	37	44.32	3.11	46.48	4.58
	SN	38	41.31	2.56	52.63	3.72
	MATH	43	35.81	3.41	56.74	4.36
	READ	43	40.00	3.27	53.95	4.13
Delayed	WR	37	21.08	2.53	14.59	2.64
	SN	38	21.84	2.41	18.42	3.05
	MATH	43	16.74	2.77	17.20	3.16
	READ	43	30.93	2.76	16.27	2.13

Note: WR – Wakeful resting (autogenic training), SN – Social networks, MATH – mathematical tasks, READ – content-similar readingpassage; N - number of participants, M_knowledge_ – mean percentual score of participants on the knowledge test, SE_knowledge_ – Standard error of the mean percentual score on the knowledge test, M_memory_ – mean percentual score of participants on the memory test, SE_memory_ – Standard error of the mean percentual score on the memory test

### Performance on the knowledge test

Performance on the knowledge test was analyzed using a repeated-measures ANOVA with percentage of correct responses as the dependent variable. There was no significant main effect of the distraction task on knowledge test performance, *F*(3, 157) = 2.56, *p* = .057, ηp^2^. = 0.05. However, there was a significant main effect of the time of the test on the performance, *F*(1, 157) = 134.88, *p* < .001, ηp^2^= 0.46, with lower performance on the delayed test. There was a significant interaction between the type of distraction task and a test timing, *F*(3, 157) = 4.05, *p* < .01, ηp^2^ = 0.07.

To further examine this interaction and test hypotheses H1a and H1b, we conducted contrast comparisons (conditional on the RM factor). First, we compared the mean of the WR condition against the average of the three other conditions at each time point. No statistically significant differences were observed, either on the immediate test (M_diff_ = 5.32, 95% C.I. = [-2.07, 12.71], t(157) = 1.42, *p* = .157, *d = 0.29*,* 95% CI [-0.11*,* 0.69])* or on the delayed test *(*M_diff_ = -2.07, 95% C.I. = [-8.29, 4.16], t(157) = -0.66, *p* = .513, *d = − 0.11*,* 95% CI [-0.45*,* 0.23]).* Next, we examined differences between the WR condition and each individual group at each time point. Regarding the percentual score of correct responses on the immediate knowledge test (H1a), we found no significant differences between groups. Specifically, no significant difference was found between the WR and SN conditions, M_diff_ = 3.01, 95% CI = [-6.10, 12.12], t(157) = 0.65, *p* = .515, *d = 0.16*,* 95% CI [-0.33*,* 0.66]*; the WR and Math conditions, M_diff_ = 8.51, 95% CI = [-0.33, 17.35], t(157) = 1.90, *p* = .059, *d = 0.46*,* 95% CI [-0.02*,* 0.94];* the WR and Read conditions, M_diff_ = 4.32, 95% CI = [-4.52, 13.17], t(157) = 0.97, *p* = .336, *d = 0.23*,* 95% CI [-0.25*,* 0.71].*

**Fig. 2:**
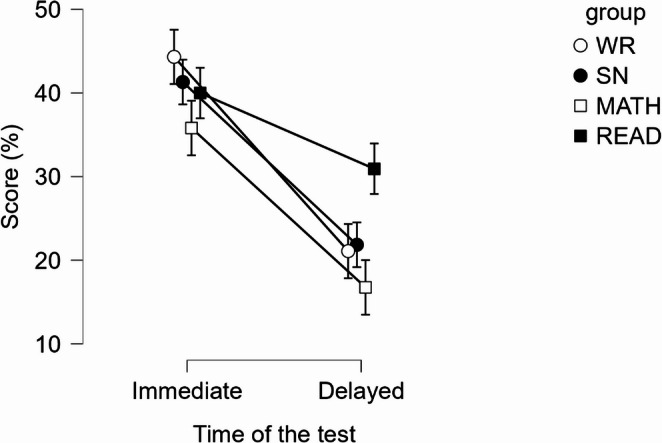
Visualization of attained scores on the immediate and delayed knowledge test

Regarding the percentual score of correct responses on the one-week delayed knowledge test (H1b), there was no significant difference found between the WR and SN conditions, M_diff_ = -0.76, 95% C.I. = [-8.44, 6.91], t(157) = -0.02, *p* = .845, *d = − 0.04*,* 95% CI [-0.46*,* 0.38];* the WR and Math conditions, M_diff_ = 4.34, 95% C.I. = [-3.11, 11.79], t(157) = 1.15, *p* = .252, *d = 0.24*,* 95% CI [-0.17*,* 0.64].* However, there was a significant difference found between the WR and Read conditions, M_diff_ = -9.85, 95% C.I. = [-17.30, -2.40], t(157) = -2.61, *p* = .010, *d = − 0.53*,* 95% CI [-0.94*,* − 0.13]*, indicating that the Read condition significantly outperformed WR condition. Results are presented in Fig. 2.

### Performance on the memory test

**Fig. 3:**
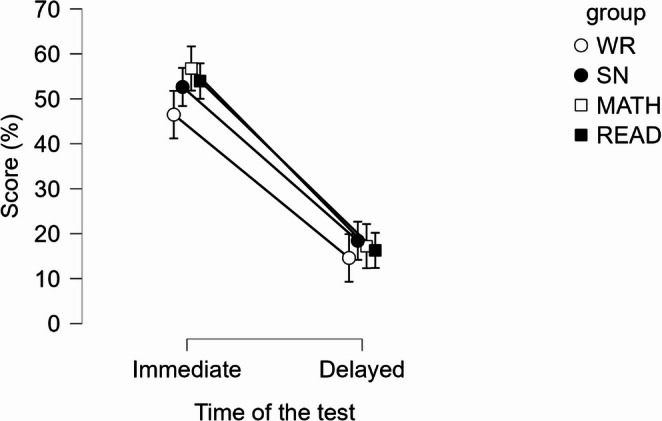
Visualization of attained scores on the immediate and delayed memory test

Performance on the memory test was analyzed using a repeated-measures ANOVA with percentage of correct responses as the dependent variable. There was no significant main effect of the type of distraction task on memory test performance, *F*(3, 157) = 0.98, *p* = .404, ηp² = 0.02. However, there was a significant main effect of time of the test, *F*(1, 157) = 246.03, *p* < .001, ηp² = 0.61, with performance declining from immediate to delayed testing. The interaction between distraction task and test timing was not significant, *F*(3, 157) = 0.56, *p* = .642, ηp² = 0.01. These findings do not support hypotheses H2a and H2b: participants in the wakeful resting condition did not significantly outperform those in the other distraction conditions (see Fig. 3).

By employing a planned contrast comparing the WR condition to the average of the remaining conditions (contrast weights: +1 for WR; −1/3 for SN, READ, and MATH), we observed that participants in the WR condition did not differ significantly from the mean of the other conditions on the immediate memory test (M_diff_ = -7.90, 95% C.I. = [-17.82, 2.02], t(157) = -1.57, *p* = .118, *d = − 0.35*,* 95% CI [-0.79*,* 0.09])*, nor the delayed memory test (M_diff_ = -2.69, 95% C.I. = [-9.21, 3.83], t(157) = -0.82, *p* = .416, *d = − 0.12*,* 95% CI [-0.41*,* 0.17]).* To further examine the differences between groups, we specified simple contrasts comparing the wakeful-rest condition with each of the other conditions. For the immediate memory test (H2a), no significant differences were found between the WR and SN conditions, M_diff_ = -6.15, 95% CI = [-18.37, 6.08], *t(157) = -0.99*,* p* = .322, *d = − 0.27*,* 95% CI [-0.81*,* 0.27];* the WR and Math conditions, M_diff_ = -10.26, 95% CI = [-22.13, 1.61], *t(157) = -1.71*,* p* = .090, *d = − 0.45*,* 95% CI [-0.98*,* 0.07] ;* or the WR and Read conditions, M_diff_ = -7.47, 95% C.I. = [-19.34, 4.40], *t(157) = -1.24*,* p* = .216, *d = − 0.33*,* 95% CI [-0.85*,* 0.20]*. For the delayed memory test (H2b), no significant differences were found between the WR and SN conditions, M_diff_ = -3.83, 95% C.I. = [-11.86, 4.21], *t(157) = − 0.94*,* p* = .348, *d = − 0.17*,* 95% CI [-0.52*,* 0.19];* the WR and Math conditions, M_diff_ = -2.62, 95% C.I. = [-10.42, 5.19], *t(157) = -0.66*,* p* = .509, *d = − 0.12*,* 95% CI [-0.46*,* 0.23];* or the WR and Read conditions, M_diff_ = -1.68, 95% C.I. = [-9.49, 6.12], *t(157) = -0.43*,* p* = .670, *d = − 0.07*,* 95% CI [-0.42*,* 0.27]*. Results are presented in Fig. 3.

## Discussion

The present study investigated the impact of different post-learning activities on comprehension and long-term retention of text material, using a reading task with ecological validity for educational contexts. Contrary to our primary hypotheses (H1a, H1b, H2a, H2b), wakeful resting (WR) implemented via autogenic training did not produce significantly better performance than post-learning activities involving social media browsing or solving math problems on either immediate or delayed knowledge and memory tests. Moreover, performance following WR did not exceed that observed in the content-similar reading condition. On the delayed knowledge test, planned contrasts revealed that participants in the reading condition performed significantly better than those in the WR condition. These findings suggest that the well-documented WR effect, primarily observed in studies focused on memorization of discrete words or facts, may not readily generalize to complex, comprehension-based learning tasks.

The absence of a consistent WR advantage in our study aligns with a growing body of literature showing inconsistent results in applied learning contexts (Brandmark et al., 2020; Leetham et al., 2024). Similar to our findings, efforts to evaluate WR in ecologically valid or real-world settings have often yielded no significant results. For example, Brandmark et al. (2020) found no significant differences in short- or long-term retention between WR and distraction conditions in a simulated classroom environment where participants watched an educational video and completed a multiple-choice knowledge test. Likewise, Leetham et al. (2024) in their online study exploring WR in naturalistic settings, found no significant effects of WR and noted that participants often struggled to remain in a truly restful state, which may have contributed to the inconclusive results. Our findings, together with findings of these studies, highlight the challenges of implementing WR protocols outside controlled laboratory environments and question the assumption that WR universally supports memory consolidation. In contrast, learning, particularly in real-world educational settings, remains a cognitively demanding process that requires effortful engagement with the material to promote durable learning (Bjork et al., 2013; Dunlosky et al., 2013). WR may benefit episodic encoding that depends heavily on hippocampal replay (Dudai et al., 2015; Schapiro et al., 2018), but comprehension tasks are likely to recruit semantic networks (Binder & Desai, 2011) that are already more integrated and less dependent on post-encoding replay.

Analyses of the distraction conditions did not reveal a consistent performance pattern across outcomes. Notably, reading a conceptually similar passage was associated with better performance than WR on the delayed knowledge test, whereas no such advantage emerged on the memory test. This selective effect suggests that post-learning reading may support the consolidation of semantic knowledge through elaborative integration (Kintsch, 1988, 1998) rather than through mechanisms supporting verbatim memory retention. In contrast, participants who solved math problems showed descriptively lower delayed performance, but this pattern was not consistent across measures and may reflect task-specific interference rather than a robust effect. Finally, although prior research has associated social media use with impaired learning and memory (Martini et al. 2020a), participants in the present study who engaged in brief, passive social media browsing did not perform significantly worse than those in the WR condition. From an interference-based perspective, this pattern suggests that not all forms of post-learning activity disrupt consolidation to the same extent. In the present design, social media use was limited to short-duration, self-paced scrolling without active interaction, which may have imposed only moderate cognitive and attentional demands. Consequently, the level of interference may have been insufficient to produce measurable consolidation costs relative to other distractor tasks.

Additionally, the pattern of performance declined from immediate to delayed testing differed between the knowledge and memory tests. Across all experimental conditions, memory test scores showed a steeper decline, dropping by roughly 30–40% points, whereas knowledge test scores declined more moderately. This divergence may reflect differences in the depth of encoding and the nature of retrieval cues involved in each test. The memory test required verbatim or surface-level recall, which is more susceptible to decay without rehearsal, whereas the knowledge test likely relied on semantic understanding, which tends to be more durable over time (Fischer & Radvansky, 2018; Kintsch, 1998).

### Limitations and Future Directions

Several conceptual and methodological limitations should be acknowledged to guide the interpretation of the present findings. Although the reading task was designed to approximate educational settings (e.g., classroom administration, time-limited reading under supervision), it did not fully capture the complexity of real classroom or self-study situations. In particular, participants were aware that the task did not carry real academic consequences (e.g., grading or course credit), and the learning context lacked features such as self-initiated study goals, extended study periods, and opportunities for strategic regulation of learning. The expository text was intentionally chosen to be novel, attractive, and easily comprehensible, yet participants may not have been equally motivated to engage deeply with unfamiliar content.

A further limitation concerns the distractor conditions. Although they were selected to represent a range of post-learning activities, they differed not only in cognitive load (e.g., math ≈ high, social media ≈ low) but also in emotional or attentional engagement (social media potentially high emotional salience, math low). These differences may partly explain the absence of a clear performance pattern across conditions. Future research should more systematically manipulate both cognitive load and emotional engagement to examine their separate and combined effects on post-learning consolidation.

Interindividual differences (e.g., baseline reading-comprehension skills, intrinsic motivation, or general cognitive ability) may also have contributed to variability in outcomes. Future research could measure these characteristics directly and statistically control for them.

Another limitation concerns the statistical precision of our estimates. Confidence intervals around group differences were often wide and included zero, reflecting a modest sample size and substantial outcome variability. This variability likely stemmed from the heterogeneity of our sample, which included students from three universities and thus encompassed a broad range of academic abilities. While this enhances ecological validity, it also increased within-group variance and reduced statistical power. Consequently, several contrasts at the delayed test remain compatible with both small benefits and small harms.

Another methodological consideration concerns the specific implementation of wakeful rest in the present study. Although autogenic training was selected to reduce mind-wandering and minimize uncontrolled rehearsal, addressing concerns raised in prior work (Varma et al., 2018; Leetham et al., 2024), it involves structured internal attention guided by verbal instructions (Schultz & Luthe, 1959). Compared to more commonly used unstructured WR paradigms (e.g., quiet sitting with closed eyes with minimal instruction, Parra et al., 2026), such guided relaxation may induce a qualitatively different cognitive state. Consequently, the present findings should not be taken to generalize to all forms of wakeful rest. Future research would benefit from directly comparing unstructured WR, guided relaxation, and mindfulness-based approaches while assessing both behavioral outcomes and physiological indicators of mental activity, to clarify whether different implementations of post-learning rest differentially support consolidation.

A further limitation concerns the measurement of learning outcomes across time. Because test versions were not counterbalanced across assessment occasions, potential test-specific effects cannot be entirely ruled out, although the two versions were designed to be comparable in content and difficulty.

Finally, our sample had an unbalanced gender ratio, which may limit the generalizability of the findings. Replication with more demographically balanced samples would strengthen the robustness of future results. In summary, these limitations point to important directions for future research, namely, further investigation of how different types of post-learning activities influence long-term learning outcomes. In particular, studies that systematically compare wakeful rest with various forms of post-learning engagement may help clarify under which conditions minimizing interference or, alternatively, promoting elaborative processing is more beneficial for learning.

### Conclusion and Implications

In conclusion, the present study contributes to the understanding of how post-learning activities influence text comprehension and retention in applied settings. Our findings suggest that wakeful resting in the form of guided relaxation may not provide clear advantages over other post-learning activities in ecologically relevant learning contexts. In fact, semantically related post-learning tasks, such as reading a similar text, may offer benefits for delayed knowledge retention. These results underscore that memory consolidation and learning as such are multifaceted phenomena, and there is no universal “magical” technique for durable learning. Rather, active engagement with learning materials appears to remain essential for achieving better comprehension and long-term retention. This interpretation is consistent with the observed advantage of the content-similar reading condition on delayed knowledge performance, which suggests that engagement with related material may have supported elaborative processing, conceptual integration, or schema construction rather than inducing purely detrimental interference (Kintsch, 1988).

## Data Availability

The research data associated with the paper is available on the link: [https://osf.io/su9j7/overview? view_only=e5e32252bfa648a6894025ed36b07584](https:/osf.io/su9j7/overview? view_only=e5e32252bfa648a6894025ed36b07584).

## Declarations

### Public Significance statement

Understanding how to best support memory after learning is important for students, teachers, and lifelong learners. This study tested whether a short period of wakeful rest after reading helps people remember and understand what they learned better than everyday activities like using social media, doing math, or reading something different. The findings suggest that wakeful rest does not reliably boost memory for complex information, highlighting that active engagement with learning material may remain key for effective learning in real educational settings.
